# Clinical analysis of 1301 children with hand and foot fractures and growth plate injuries

**DOI:** 10.1186/s12891-024-07407-z

**Published:** 2024-04-08

**Authors:** Tianfeng Zhu, Xin Qiu, Hansheng Deng, Haoran Feng, Jianlin Chen, Zilong Huang, Jiahui Li, Shizhe Liu, Shuaiyin Wang, Zhenkun Gu, Zhengyu Wu, Qisong Yang, Gen Liu, Leonardo Antonio Sechi, Gianfilippo Caggiari, Chao You, Guibing Fu

**Affiliations:** 1 Shenzhen Children’s Hospital, Shenzhen, People’s Republic of China; 2 Shenzhen Pediatrics Institute of Shantou University Medical College, Shantou, People’s Republic of China; 3 Clinical research center, Hefei cancer hospital, Chinese academy of sciences, Hefei, People’s Republic of China; 4 Hefei center for disease control and prevention, Hefei, People’s Republic of China; 5 Department of Biomedical Sciences, University of Sassari, 07100 Sassari, Italy; 6 Orthopaedic Department, Sassari University Hospital, 07100 Sassari, Italy

**Keywords:** Children, Hands, Feet, Fracture sites, Etiologies

## Abstract

**Background:**

Fractures of hands and feet are common in children, but relevant epidemiological studies are currently lacking. We aim to study the epidemiological characteristics of hand and foot fractures and growth plate injuries in children and provide a theoretical basis for their prevention, diagnosis, and treatment.

**Methods:**

We retrospectively analyzed the data of children with hand and foot fractures who were hospitalized at Shenzhen Children’s Hospital between July 2015 and December 2020. Data on demographic characteristics, fracture site, treatment method, etiology of injury, and accompanying injuries were collected. The children were divided into four age groups: infants, preschool children, school children, and adolescents. The fracture sites were classified as first-level (the first–fifth finger/toe, metacarpal, metatarsal, carpal, and tarsal) and second-level (the first–fifth: proximal phalanx, middle phalanx, distal phalanx, metacarpal, and metatarsal) sites. The changing trends in fracture locations and injury causes among children in each age group were analyzed.

**Results:**

Overall, 1301 children (1561 fractures; 835 boys and 466 girls) were included. The largest number of fractures occurred in preschool children (*n* = 549, 42.20%), with the distal phalanx of the third finger being the most common site (*n* = 73, 15.57%). The number of fractures in adolescents was the lowest (*n* = 158, 12.14%), and the most common fracture site was the proximal phalanx of the fifth finger (*n* = 45, 29.61%). Of the 1561 fractures, 1143 occurred in the hands and 418 in the feet. The most and least common first-level fracture sites among hand fractures were the fifth (*n* = 300, 26.25%) and first (*n* = 138, 12.07%) fingers, respectively. The most and least common first-level foot fracture locations were the first (*n* = 83, 19.86%) and fourth (*n* = 26, 6.22%) toes, respectively. The most common first-level and second level etiologies were life related injuries (*n* = 1128, 86.70%) and clipping injuries (*n* = 428, 32.90%), respectively. The incidence of sports injuries gradually increased with age, accounting for the highest proportion in adolescents (26.58%). Hand and foot fractures had many accompanying injuries, with the top three being nail bed injuries (570 cases, 36.52%), growth plate injuries (296 cases, 18.96%), and distal severed fracture (167 cases, 10.70%). Among the 296 growth plate injuries, 246 occurred on the hands and 50 on the feet.

**Conclusions:**

In contrast to previous epidemiological studies on pediatric hand and foot fractures, we mapped the locations of these fractures, including proximal, shaft, distal, and epiphyseal plate injuries. We analyzed the changing trends in fracture sites and injury etiologies with age. Hand and foot fractures have many accompanying injuries that require attention during diagnosis and treatment. Doctors should formulate accident protection measures for children of different ages, strengthen safety education, and reduce the occurrence of accidental injuries.

## Background

The global population is constantly increasing. According to data from the United Nations Population Division, the global population currently exceeds 8 billion, of which children account for a large proportion [1]. Compared to an adult, a child’s physical and mental development is immature. Children lack the perception of danger, therefore, they are vulnerable to accidental injuries during daily activities [2]. Among these accidental injuries, fractures of the hands and feet are relatively common. According to Alfort’s report, hand fractures are the most common fractures in the upper limbs, and even simple cases can lead to children being unable to play normally for several months [3]. Foot fracture constitutes about 32.4% of all lower limb fractures and affects both physical and mental health, the incidence of foot fracture is higher than that of humerus, clavicle and patella [4]. And their adverse effects on children require attention from doctors and parents.

Studies have reported that hand injuries account for a high proportion of pediatric emergency department visits [5]. Hand fractures are the second most common fractures in children, accounting for 20% of all cases of childhood fractures [6, 7]. In addition, foot fractures account for 8% of all fractures in children [6, 8]. Hand fractures, especially in the fingers, can significantly impact delicate hand movements and seriously affect a child’s life, learning, and play. In addition, because the foot plays a weight-bearing role, foot fractures affect the gait and movement of the lower limbs [9]. Therefore, the tolerance for residual deformity in hand and foot fractures is lower than that for other fractures [10]. If hand and foot fractures are not properly treated, and deformities remain, severe consequences can occur throughout a child’s life [11]. Due to the high incidence of hand and foot fractures in children and the adverse impacts on children, therefore our objective is to research the epidemiological characteristics of hand and foot fractures in children and provide reference for the prevention.

Although some studies have evaluated hand fractures in children, they lack details on fracture sites, and include only a limited number of cases. Moreover, epidemiological studies on foot fractures have rarely been reported. Therefore, in this study, we aimed to go a step further by specifically locating the hand and foot fracture sites to the proximal, shaft, and distal portions to document the distribution and characteristics of hand and foot fractures in children. In addition, we collected information on etiology and accompanying injuries. We studied the patterns of hand and foot fractures in children to provide a reference for reducing accidental injuries and diagnosing and treating hand and foot fractures.

## Methods

We retrospectively analyzed children with hand and foot fractures who were hospitalized at the Shenzhen Children’s Hospital between July 2015 and February 2020. The inclusion criteria were as follows: 1. hand and foot fractures in children under 18 years of age and 2. fresh fractures that occurred within 14 days. The exclusion criteria were as follows: 1. pathological fractures and 2. combined fractures at other locations. From the medical records, data on sex, age, etiology, fracture site, treatment method, anesthesia method, hospitalization stay, hospital charges, and accompanying injuries were obtained. All of the procedures were in accordance with the ethical standards of the national committees on human experimentation and the Helsinki Declaration of 1964 and later versions and approved by the Medical Ethics Committee of the Shenzhen Children’s Hospital (Ref number:2020091).

Inpatients were grouped by age: age ≤ 2 y, infants; 2 y < age ≤ 6 y, preschool children; 6 y < age ≤ 11 y, school children; and 11 y < age ≤ 18 y, adolescents. Referring to previous studies [12, 13], we divided the etiologies of the injuries into five first-level and 29 second-level etiologies. First-level etiologies included injuries of daily life, road traffic, sports, abuse, and unknown causes. Second-level etiologies were as follows: daily life injuries included falls, crush injuries, clipping, furniture-related falls, sprains, bunk bed falls, falls from heights, cuts, strains, and twist injuries. Road traffic injuries were divided into car accident injuries, bicycle falls, bike-spoke injuries, and falls from vehicles. Sports injuries were divided into single or parallel bar falls, falls while running, kick injuries, falls during physical education activities, as well as skateboard, soccer, basketball, trampoline, balance bike, dance, slide, ice-skating, playground, taekwondo, and jump-rope falls.

To better describe the fracture sites, we divided them into first- and second-level sites. First-level fracture sites included the first–fifth finger/toe, metacarpal, metatarsal, carpal, and tarsal. Second-level fracture sites included the first–fifth: proximal phalanx, middle phalanx, distal phalanx, metacarpal, and metatarsal. In addition, we subdivided tubular bone fractures into proximal, shaft, and distal portion fractures to further study the epidemiological characteristics of hand and foot fractures in children. Finally, we created the graph of the distribution of fractures to illustrate these results clearly.

## Results

### Demographic information

A total of 1301 children (1561 fractures), including 835 boys and 466 girls at a male-to-female ratio of 1.79:1, were included in the study (Table 1). Among infants, preschool children, school children, and adolescents, males accounted for 52, 63, 65, and 88% of participants, respectively.

**Table 1:** Demographics of patients with 1301 patients(1561 fracture sites)

Parameter	Patients n(%)
Numbers	1301
Age class	
Infants	247(18.99%)
Preschool children	549(42.20%)
School children	347(26.67%)
Adolescents	158(12.14%)
Sex	
Male	835(64.18%)
Female	466(35.82%)
Side	
Left	774(48.58%)
Right	787(50.42%)
Hospitalization expenses (RMB)	6214.35(IQR,4248.71–7378.96)yuan
Hospital stays (day)	3.79 ± 2.90

Among the 1561 fractures, a total of 774 fractures occurred on the left side and 787 on the right side. The largest number of fractures occurred in preschool children (*n* = 549, 42.20%), and the smallest number of fractures occurred in adolescents (*n* = 158, 12.14%). Overall, the average hospital stay was 3.79 ± 2.90 days, and the average hospitalization expense was 6214.35 yuan (interquartile range: 4248.71–7378.96 yuan) (Table 1).

### Fracture sites of the hand

**Fig. 1:**
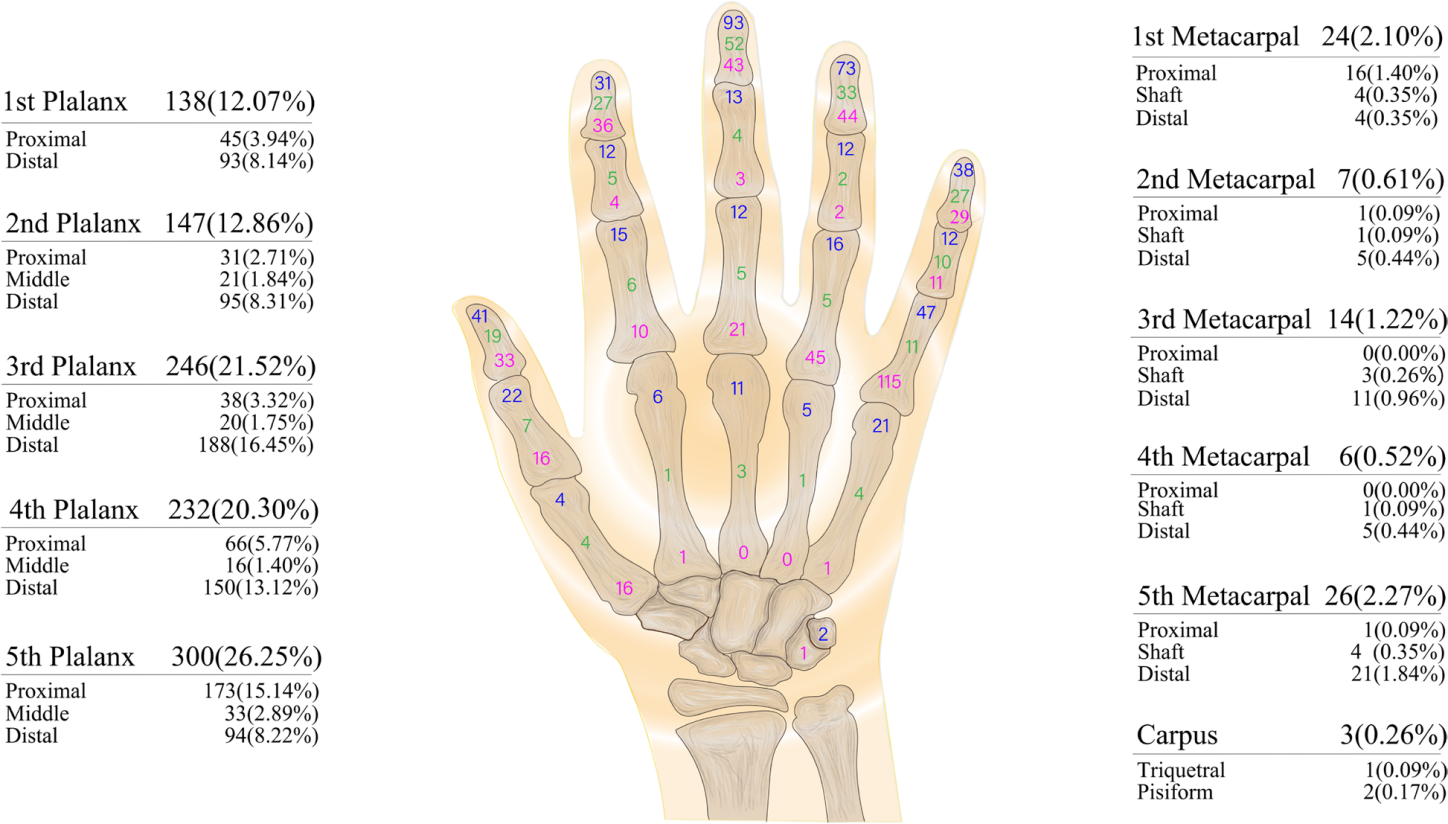
Detailed sites of 1143 cases of hand fractures. The number of proximal, shaft, and distal portion fractures for each long bone is marked in the corresponding area, and the total number and percentage of fractures for each finger is marked on the side of the figure

Among the 1561 fractures, 1143 (73.22%) occurred in the hands, with the most occurring in preschool children (*n* = 469, 41.03%), and the fewest in adolescents (*n* = 152, 13.30%). The most common first-level fracture site in preschool children, school children, and adolescents was the fifth finger, whereas the most common site in infants was the third finger. In the four age groups, 300 fractures occurred in the fifth finger (26.25%), accounting for the largest proportion of first-level fracture sites (Table 2 and Fig. 1).

**Table 2:** The epidemiology of age group according to different hand fractures sites

Fractures sites	Infants		Preschool children		School children		Adolescents		Total	
	No.	%	No.	%	No.	%	No.	%	No.	%
1st phalanx	32	13.68%	72	15.35%	22	7.64%	12	7.89%	138	12.07%
Proximal phalanx	7	2.99%	19	4.05%	10	3.47%	9	5.92%	45	3.94%
Distal phalanx	25	10.68%	53	11.30%	12	4.17%	3	1.97%	93	8.14%
2nd phalanx	34	14.53%	70	14.93%	34	11.81%	9	5.92%	147	12.86%
Proximal phalanx	5	2.14%	11	2.35%	10	3.47%	5	3.29%	31	2.71%
Middle phalanx	7	2.99%	9	1.92%	3	1.04%	2	1.32%	21	1.84%
Distal phalanx	22	9.40%	50	10.66%	21	7.29%	2	1.32%	95	8.31%
3rd phalanx	67	28.63%	90	19.19%	73	25.35%	16	10.53%	246	21.52%
Proximal phalanx	3	1.28%	12	2.56%	14	4.86%	9	5.92%	38	3.32%
Middle phalanx	9	3.85%	5	1.07%	4	1.39%	2	1.32%	20	1.75%
Distal phalanx	55	23.50%	73	15.57%	55	19.10%	5	3.29%	188	16.45%
4th phalanx	62	26.50%	90	19.19%	54	18.75%	26	17.11%	232	20.30%
Proximal phalanx	6	2.56%	12	2.56%	28	9.72%	20	13.16%	66	5.77%
Middle phalanx	3	1.28%	6	1.28%	4	1.39%	3	1.97%	16	1.40%
Distal phalanx	53	22.65%	72	15.35%	22	7.64%	3	1.97%	150	13.12%
5th phalanx	32	13.68%	125	26.65%	90	31.25%	53	34.87%	300	26.25%
Proximal phalanx	4	1.71%	59	12.58%	65	22.57%	45	29.61%	173	15.14%
Middle phalanx	5	2.14%	17	3.62%	7	2.43%	4	2.63%	33	2.89%
Distal phalanx	23	9.83%	49	10.45%	18	6.25%	4	2.63%	94	8.22%
Metacarpal	5	2.14%	22	4.69%	14	4.86%	36	23.68%	77	6.74%
1st metacarpal	1	0.43%	6	1.28%	9	3.13%	8	5.26%	24	2.10%
2nd metacarpal	1	0.43%	5	1.07%	0	0.00%	1	0.66%	7	0.61%
3rd metacarpal	2	0.85%	7	1.49%	1	0.35%	4	2.63%	14	1.22%
4th metacarpal	0	0.00%	1	0.21%	0	0.00%	5	3.29%	6	0.52%
5th metacarpal	1	0.43%	3	0.64%	4	1.39%	18	11.84%	26	2.27%
Carpus	2	0.85%	0	0.00%	1	0.35%	0	0.00%	3	0.26%
Total	234	100.00%	469	100%	288	100.00%	152	100.00%	1143	100.00%

The three most common second-level fracture sites were the distal phalanx of the third finger (*n* = 188, 16.45%), proximal portion of the fifth finger (*n* = 173, 15.14%), and distal phalanx of the fourth finger (*n* = 150, 13.12%). In infants and preschool children, the distal phalanx of the third finger was the most common second-level fracture site (55, 23.50% and 73, 15.57%, respectively). In school children and adolescents, the proximal phalanx of the fifth finger was the most common second-level fracture site (65, 22.57% and 45, 29.61%, respectively) (Table 2 and Fig. 1).

Data for all age groups showed that the number of phalanx fractures was the highest (*n* = 1063), followed by metacarpal (*n* = 77) and carpal fractures (*n* = 3). The number of fractures at the proximal portion of proximal phalanx of the fifth finger and distal portion of the fifth metacarpal was 136, accounting for 41.72% of fifth-line fractures (Table 2 and Fig. 1).

Considering the essential functions of the first and second fingers, we calculated the number and proportion of fractures that occurred in the first and second fingers among all finger fractures. In infants, 66 fractures occurred in the first and second fingers, accounting for 29.07% of the total number of finger fractures. There were 142 (31.77%), 56 (20.51%), and 21 (18.10%) fractures in the first and second fingers in preschool children, school children, and adolescents, respectively (Table 2 and Fig. 1).

### Fracture sites of the foot

**Fig. 2:**
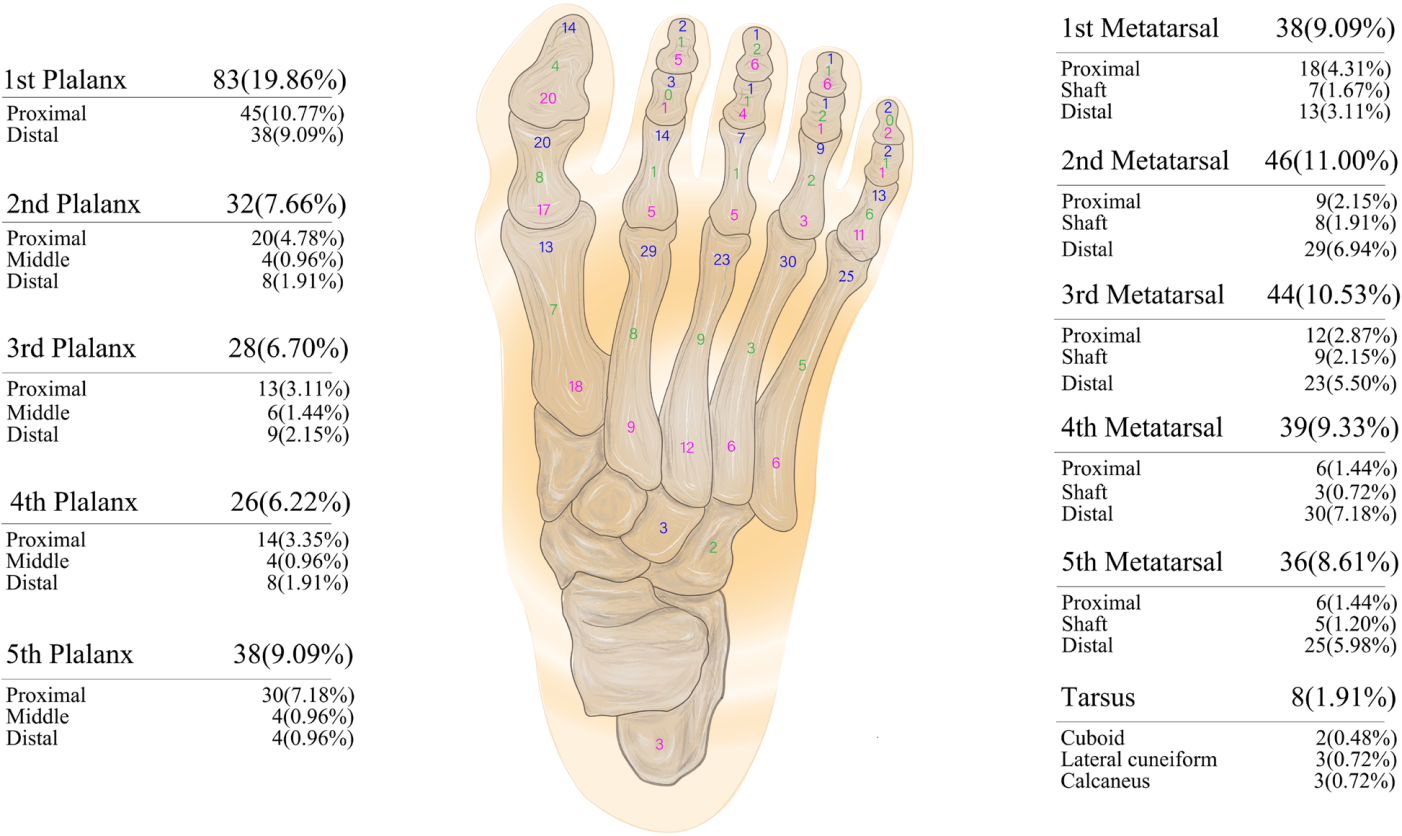
Detailed sites of 418 cases of foot fractures. The number of proximal, shaft, and distal portion fractures of each long bone is marked in the corresponding area, and the total number and percentage of fractures of each toe is marked on the side of the figure

Among the 1561 fractures, a total of 418 (26.78%) fractures occurred in the feet. Among them, the number of fractures in preschool children was the highest (*n* = 177, 42.34%), and that in adolescents was the lowest (*n* = 43, 10.29%) (Table 3 and Fig. 2). This result is similar to that for hand fractures.

**Table 3:** The epidemiology of age group according to different foot fractures sites

Fractures sites	Infants		Preschool children		School children		Adolescents		Total	
	No.	%	No.	%	No.	%	No.	%	No.	%
1st phalanx	17	32.69%	42	23.73%	18	12.33%	6	13.95%	83	19.86%
Proximal phalanx	7	13.46%	21	11.86%	12	8.22%	5	11.63%	45	10.77%
Distal phalanx	10	19.23%	21	11.86%	6	4.11%	1	2.33%	38	9.09%
2nd phalanx	4	7.69%	8	4.52%	18	12.33%	2	4.65%	32	7.66%
Proximal phalanx	2	3.85%	5	2.82%	13	8.90%	0	0.00%	20	4.78%
Middle phalanx	1	1.92%	1	0.56%	1	0.68%	1	2.33%	4	0.96%
Distal phalanx	1	1.92%	2	1.13%	4	2.74%	1	2.33%	8	1.91%
3rd phalanx	7	13.46%	11	6.21%	10	6.85%	0	0.00%	28	6.70%
Proximal phalanx	2	3.85%	4	2.26%	7	4.79%	0	0.00%	13	3.11%
Middle phalanx	2	3.85%	3	1.69%	1	0.68%	0	0.00%	6	1.44%
Distal phalanx	3	5.77%	4	2.26%	2	1.37%	0	0.00%	9	2.15%
4th phalanx	2	3.85%	12	6.78%	12	8.22%	1	2.33%	26	6.22%
Proximal phalanx	1	1.92%	4	2.26%	9	6.16%	1	2.33%	14	3.35%
Middle phalanx	0	0.00%	2	1.13%	2	1.37%	0	0.00%	4	0.96%
Distal phalanx	1	1.92%	6	3.39%	1	0.68%	0	0.00%	8	1.91%
5th phalanx	4	7.69%	19	10.73%	14	9.59%	1	2.33%	38	9.09%
Proximal phalanx	2	3.85%	16	9.04%	11	7.53%	1	2.33%	30	7.18%
Middle phalanx	1	1.92%	0	0.00%	3	2.05%	0	0.00%	4	0.96%
Distal phalanx	1	1.92%	3	1.69%	0	0.00%	0	0.00%	4	0.96%
Metatarsal	18	34.62%	81	45.76%	71	48.63%	33	76.74%	203	48.56%
1st metatarsal	5	9.62%	20	11.30%	10	6.85%	3	6.98%	38	9.09%
2nd metatarsal	3	5.77%	16	9.04%	18	12.33%	9	20.93%	46	11.00%
3rd metatarsal	4	7.69%	15	8.47%	17	11.64%	8	18.60%	44	10.53%
4th metatarsal	3	5.77%	15	8.47%	14	9.59%	7	16.28%	39	9.33%
5th metatarsal	3	5.77%	15	8.47%	12	8.22%	6	13.95%	36	8.61%
Tarsus	0	0.00%	4	2.26%	3	2.05%	1	2.33%	8	1.91%
Total	52	100.00%	177	100.00%	146	100.00%	43	100.00%	418	100.00%

The most common first-level fracture site in infants, preschool children, school children, and adolescents was the metatarsal (*n* = 203, 48.56%). Among the five toes, the most common fracture site was the first toe (*n* = 83, 19.86%) and the least common was the fourth toe (*n* = 26, 6.22%) (Table 3 and Fig. 2).

The top 3 second-level fracture sites were the second metatarsal (*n* = 46, 11.00%), proximal phalanx of the first toe (*n* = 45, 10.77%), and third metatarsal (*n* = 44, 10.53%). In infants, the distal phalanx of the first toe was the most common second-level fracture site (*n* = 10; 19.23%). In preschool children, the most common second-level fracture sites were the proximal and distal phalanges of the first toe (*n* = 21 each, 11.86%). Data from school children and adolescents showed that the second metatarsal was the most common second-level fracture site in these age groups (*n* = 18, 12.23% and *n* = 9, 20.93%, respectively) (Table 3 and Fig. 2).

Among the 418 ft fractures, the number of phalanx fractures was the highest (*n* = 207), followed by metatarsal (*n* = 203) and tarsal (*n* = 8) fractures (Table 3 and Fig. 2).

### Etiologies of the injuries

**Fig. 3:**
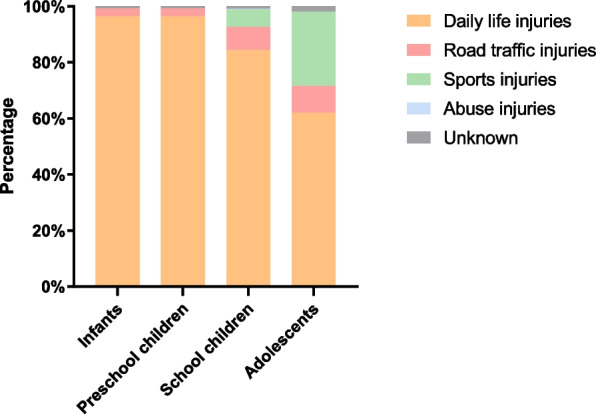
Causes of injury in different age groups

For all children, the most common first-level etiology was daily life injury (1128 children, 86.70%) and the least common was injury from abuse (1 child, 0.08%) (Table 4, Fig. 3). The most common second-level etiology was clipping (428 children, 32.90%), followed by falls (257 children, 19.75%), crushing injuries (248 children, 19.06%), and cuts (138 children, 10.61%) (Table 4).

**Table 4:** The epidemiology of age group according to different etiologies

Causes of fractures	Infants		Preschool children		School children		Adolescents		Total	
	No.	%	No.	%	No.	%	No.	%	No.	%
Daily life injuries	238	96.36%	499	90.89%	293	84.44%	98	62.03%	1128	86.70%
Falls	7	2.83%	69	12.57%	114	32.85%	67	42.41%	257	19.75%
Crush injuries	63	25.51%	112	20.40%	62	17.87%	11	6.96%	248	19.06%
Clipping	124	50.20%	222	40.44%	71	20.46%	11	6.96%	428	32.90%
Furniture-related falls	0	0.00%	7	1.28%	1	0.29%	1	0.63%	9	0.69%
Sprains	1	0.40%	3	0.55%	2	0.58%	2	1.27%	8	0.61%
Bunk bed falls	0	0.00%	9	1.64%	0	0.00%	0	0.00%	9	0.69%
Falls from height	0	0.00%	1	0.18%	2	0.58%	1	0.63%	4	0.31%
Cuts	31	12.55%	64	11.66%	38	10.95%	5	3.16%	138	10.61%
Strains	0	0.00%	0	0.00%	1	0.29%	0	0.00%	1	0.08%
Twist injuries	12	4.86%	12	2.19%	2	0.58%	0	0.00%	26	2.00%
Road traffic injuries	7	2.83%	35	6.38%	28	8.07%	15	9.49%	85	6.53%
Car accident injuries	6	2.43%	21	3.83%	21	6.05%	9	5.70%	57	4.38%
Bicycle falls	0	0.00%	0	0.00%	3	0.86%	5	3.16%	8	0.61%
Bicycle-spoke injuries	1	0.40%	14	2.55%	4	1.15%	1	0.63%	20	1.54%
Falls from vehicles	0	0.00%	0	0.00%	0	0.00%	0	0.00%	0	0.00%
Sports injuries	0	0.00%	10	1.82%	22	6.34%	42	26.58%	74	5.69%
Single/parallel bar falls	0	0.00%	0	0.00%	0	0.00%	0	0.00%	0	0.00%
Basketball falls	0	0.00%	1	0.18%	7	2.02%	27	17.09%	35	2.69%
Falls while running	0	0.00%	2	0.36%	1	0.29%	1	0.63%	4	0.31%
Skateboard falls	0	0.00%	0	0.00%	2	0.58%	1	0.63%	3	0.23%
Soccer falls	0	0.00%	1	0.18%	3	0.86%	4	2.53%	8	0.61%
Kick injuries	0	0.00%	0	0.00%	6	1.73%	7	4.43%	13	1.00%
Falls during physical education activities	0	0.00%	0	0.00%	0	0.00%	1	0.63%	1	0.08%
Trampoline falls	0	0.00%	0	0.00%	1	0.29%	0	0.00%	1	0.08%
Balance bike falls	0	0.00%	0	0.00%	0	0.00%	0	0.00%	0	0.00%
Dance falls	0	0.00%	0	0.00%	1	0.29%	0	0.00%	1	0.08%
Slide falls	0	0.00%	3	0.55%	0	0.00%	0	0.00%	3	0.23%
Ice skating falls	0	0.00%	1	0.18%	0	0.00%	0	0.00%	1	0.08%
Playground falls	0	0.00%	2	0.36%	0	0.00%	0	0.00%	2	0.15%
Taekwondo falls	0	0.00%	0	0.00%	1	0.29%	1	0.63%	2	0.15%
Jump rope falls	0	0.00%	0	0.00%	0	0.00%	0	0.00%	0	0.00%
Abuse injuries	0	0.00%	0	0.00%	1	0.29%	0	0.00%	1	0.08%
Unknown	2	0.81%	5	0.91%	3	0.86%	3	1.90%	13	1.00%
Total	247	100.00%	549	100.00%	347	100.00%	158	100.00%	1301	100.00%

The etiologies of the injuries differed in children of different ages. Among the infants, 238 (96.36%) suffered hand and foot fractures due to daily life injuries (Table 4, Fig. 3). Among these, 124 (50.20%) had clipping injuries, 63(25.51%) had crush injuries, and 31 (12.55%) had cuts. No sports-related injuries occurred in the infants (Table 4).

A total of 499 (90.89%) preschool children suffered injuries in daily life (Table 4, Fig. 3). Among these, 222(40.44%) had clipping injuries, 112(20.40%) had crush injuries, 69(12.57%) had falls, and 31(11.66%) had cuts. Preschool children began to experience sports injuries, but only 10 (1.82%) did (Table 4).

In school children, the incidence of daily-life injuries decreased, whereas that of sports injuries increased. A total of 293 (84.44%) school children suffered daily-life injuries (Table 4, Fig. 3). Among them, 114 experienced falls (32.85%), 71 had clipping injuries (20.46%), 62 had crush injuries (17.87%), and 38 had cuts (10.95%). A total of 22 (6.34%) children had sports injuries (Table 4).

The etiologies of injuries varied significantly among adolescents. Compared to other age groups, the incidence of daily-life injuries in adolescents relatively reduced, whereas that of sports injuries increased significantly. There were 98 (62.03%) cases of daily life injuries in adolescents, including 67 cases of falls (42.41%), 11 of crush injuries (6.96%), 11 of clipping injuries (6.96%), and 5 of cuts (3.16%). Moreover, 42 (26.58%) cases of sports injuries were observed, of which basketball falls were the most common (27, 17.09%)(Table 4, Fig. 3).

### Treatment

Most hand and foot fractures undergo closed reduction, open reduction is uncommon. Thenar flap transfer is mainly used in children with distal severed injuries that may be completely or partially severed but with poor blood supply. In addition, because children’s bones are softer than those of adults, suture internal fixation is also an option. Among the 1561 fractures in this study, 525 (33.63%) were treated with open reduction and 897 (57.46%) with closed reduction. The closed-to-open reduction ratio was 1.71:1 (Table 5).

**Table 5:** Treatment and anesthesia

Parameter	Patients
Open reduction subgroup	525(33.63%)
Kirschner wire fixation	453(29.02%)
Suture fixation	71(4.55%)
Screw fixation	1(0.06%)
Closed reduction subgroup	897(57.46%)
Kirschner wire fixation	524(33.57%)
Plaster fixation	371(23.77%)
Screw fixation	2(0.13%)
Thenar flap transfer subgroup	70(4.48%)
Unknow treatment subgroup	69(4.42%)
Local anesthesia	83(6.38%)
General anesthesia	1218(93.62%)

Among the fractures treated with open reduction, Kirschner wires were used in 453 cases, suture internal fixation was used in 71 cases, and screws were used in one case. Among the fractures that were treated with closed reduction, Kirschner wires were used in 524 cases, plaster external fixation in 371 cases, and screws in two cases. In addition, 70 (4.48%) patients underwent thenar flap transfer because of distal severed injuries, and the treatment method for 69 patients (4.42%) was unknown. Regarding the anesthesia method, 1218 patients were administered general anesthesia, whereas only 83 were administered local anesthesia (Table 5).

### Accompanying injuries

In the study, hand and foot fractures were accompanied by 1319 injuries, including nail bed (570 cases, 43.21%), growth plate (296 cases, 22.44%), distal severed (167 cases, 12.66%), nerve (24 cases, 1.82%), vascular (37 cases, 2.81%), tendon (145 cases, 10.99%), and joint capsule (50 cases, 3.79%) injuries, as well as compartment syndrome (30 cases, 2.27%) (Table 6).

**Table 6:** Accompanying injuries

Accompanying injuries	Number
Nail bed injury	570(43.21%)
Epiphyseal plate injury	296(22.44%)
Distal severed injury	167(12.66%)
Complete severed injury	16(1.21%)
Partial severed injury	151(11.45%)
Nerve injury	24(1.82%)
Finger	19(1.44%)
Toe	5(0.38%)
Vascular injury	37(2.81%)
Finger	26(1.97%)
Toe	11(0.84%)
Tendon injury	145(10.99%)
Finger	82(6.22%)(Flexor tendon19, Extensor tendon63)
Toe	63(4.78%)(Flexor tendon22, Extensor tendon41)
Joint capsular injury	50(3.79%)
Finger	34(2.58%)
Toe	16(1.21%)
Compartment syndrome	30(2.27%)

Among the 167 cases of distal severed injuries, 16 were complete and 151 were partial. Sixteen children with complete distal severed injuries were treated with thenar skin flap transfer. Among the 151 children with partial distal severed injuries, 54 also underwent thenar flap transfer surgery owing to poor blood supply to the distal finger (Table 6).

Among the cases of nerve injuries, 19 involved damage to the proper nerves of the fingers and five involved damage to the proper nerves of the toes. Among the cases of vascular injuries, 26 involved damage to the proper blood vessels of the fingers and 11 involved damage to the proper blood vessels of the toes. Furthermore, among tendon injuries, extensor tendon injuries were more prevalent than flexor tendon injuries. There were 63 and 41 cases in the extensor digital tendons of the fingers and toes, respectively, and 19 and 22 cases in the flexor digital tendons of the fingers and toes, respectively. Notably, 30 cases of compartment syndrome were observed, with an incidence rate of 1.72%. Among these, 12 cases occurred in the hands and 18 in the feet (Table 6).

### Growth plate injuries

**Fig. 4:**
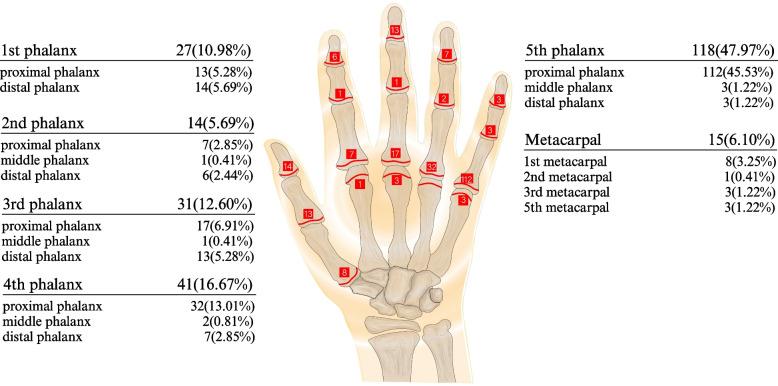
The distribution of growth plate injuries in hand. The red line represents the growth plate of hand, and the number in the adjacent red box represents the number of growth plate injuries

**Fig. 5:**
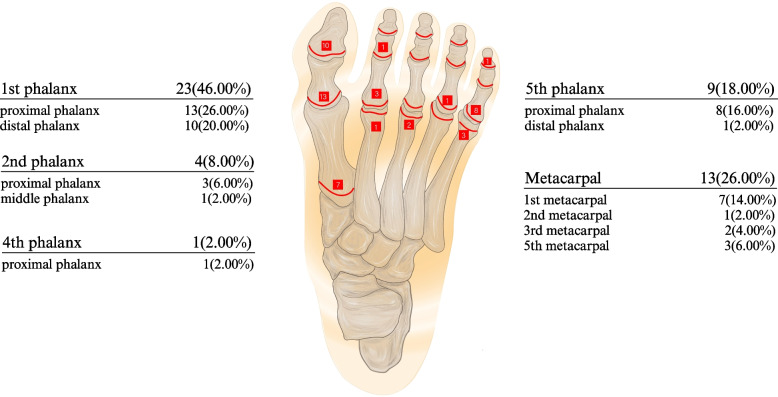
The distribution of growth plate injuries in foot. The red line represents the growth plate of foot, and the number in the adjacent red box represents the number of growth plate injuries

Among the 296 cases of growth plate injuries, 246 occurred in the hands and 50 in the feet. Among the growth plate injuries of the hand, the fifth finger was the most common (*n* = 118) and the second finger was the least common (*n* = 14) site. There were 112 cases of growth plate injuries at the proximal phalanx of the fifth finger and three at the fifth metacarpal, equaling 115. A total of 136 fractures occurred near the fifth metacarpophalangeal joint, of which the proportion of growth plate injuries was as high as 84.56%. Among the growth plate injuries of the foot, the first toe was the most common (*n* = 23) and the third toe was the least common (*n* = 0) site. There were 25 cases of growth plate injuries in the proximal phalanx, 11 in the distal phalanx, and one in the middle phalanx (Table 7, Figs. 4 and 5).

**Table 7:** Distribution of growth plate injuries

Hand/Foot	Fracture site	No.	%
Hand	Metacarpal	15	6.10%
	Proximal portion of 1st metacarpal	8	3.25%
	Distal portion of 2nd metacarpal	1	0.41%
	Distal portion of 3rd metacarpal	3	1.22%
	Distal portion of 5th metacarpal	3	1.22%
	1st phalanx	27	10.98%
	Proximal portion of the proximal phalanx of 1st finger	13	5.28%
	Proximal portion of the distal phalanx of 1st finger	14	5.69%
	2nd phalanx	14	5.69%
	Proximal portion of the proximal phalanx of 2nd finger	7	2.85%
	Proximal portion of the distal phalanx of 2nd finger	6	2.44%
	Proximal portion of the middle phalanx of 2nd finger	1	0.41%
	3rd phalanx	31	12.60%
	Proximal portion of the proximal phalanx of 3rd finger	17	6.91%
	Proximal portion of the distal phalanx of 3rd finger	13	5.28%
	Proximal portion of the middle phalanx of 3rd finger	1	0.41%
	4th phalanx	41	16.67%
	Proximal portion of the proximal phalanx of 4th finger	32	13.01%
	Proximal portion of the distal phalanx of 4th finger	7	2.85%
	Proximal portion of the middle phalanx of 4th finger	2	0.81%
	5th phalanx	118	47.97%
	Proximal portion of the proximal phalanx of 5th finger	112	45.53%
	Proximal portion of the distal phalanx of 5th finger	3	1.22%
	Proximal portion of the middle phalanx of 5th finger	3	1.22%
	Total	246	100.00%
Foot	Metatarsal	13	26.00%
	Proximal portion of 1st Metatarsal	7	14.00%
	Distal portion of 2nd Metatarsal	1	2.00%
	Distal portion of 3rd Metatarsal	2	4.00%
	Distal portion of 5th Metatarsal	3	6.00%
	1st phalanx	23	46.00%
	Proximal portion of the proximal phalanx of 1st finger	13	26.00%
	Proximal portion of the distal phalanx of 1st finger	10	20.00%
	2nd phalanx	4	8.00%
	Proximal portion of the proximal phalanx of 2nd finger	3	6.00%
	Proximal portion of the middle phalanx of 2nd finger	1	2.00%
	4th phalanx	1	2.00%
	Proximal portion of the proximal phalanx of 4th finger	1	2.00%
	5th phalanx	9	18.00%
	Proximal portion of the proximal phalanx of 5th finger	8	16.00%
	Proximal portion of the distal phalanx of 5th finger	1	2.00%
	Total	50	100.00%

## Discussion

Overall, this study reveals some key findings regarding hand and foot fractures in children. The key findings of the study are as follows: First, the highest incidence of hand and foot fractures was observed in preschool children. Second, among hand fractures, the most common first-level fracture site was the fifth finger, and the most common second-level fracture site was the distal phalanx of the third finger. Third, among foot fractures, the most common first-level fracture site was the metatarsal, and the most common second-level fracture site was the second metatarsal. Fourth, most injuries were caused by daily life injuries, of which clipping injuries were the most common. Compared to other age groups, sports injuries were the most prevalent in adolescents. Finally, hand and foot fractures often had multiple accompanying injuries, including nail bed, growth plate, and distal severed injuries.

These findings suggest that it is crucial to implement appropriate accidental injury prevention measures and provide family safety education based on the common locations and etiologies of injuries to the children’s hands and feet. In addition, orthopedic surgeons should consider the diagnosis and treatment of accompanying injuries to improve the prognosis of children with hand and foot fractures.

### Sex and age

Several epidemiological studies on hand fractures in children have reported that these types of fractures are more common in boys [14–16]. Consistent with previous studies, the results of this study showed that more male patients were affected in each age group. The differences between males and females were the smallest in infants and gradually increased. This difference was the most significant in adolescents, where male patients were approximately seven times more likely to be affected than female patients. This result may be explained by males having higher androgen levels, greater strength, and a greater tendency to exercise [17, 18]. Consequently, males are more likely to be injured during daily life and sports activities.

A prospective study of isolated finger injuries in children by Doraiswamy et al. [19] showed that finger fractures were the most common in children younger than 5 years old. However, other scholars believe the incidence is higher in adolescents [14, 16]. In this study, the highest number of fractures occurred in preschool children and the lowest occurred in adolescents. Preschool children have recently acquired the skills to walk and run, beginning to develop the ability to move independently. Simultaneously, they are curious about the world around them and constantly want to explore new things; however, they have a weak sense of self-protection and are, therefore, vulnerable to accidental harm [2]. Adolescents are in a transition stage from childhood to adulthood. During their physical and psychological development, they have social and psychological characteristics that differ significantly from those of younger children. Therefore, they are more aware of dangers and are less likely to be injured [20].

### Fractures of the hand

Multiple studies have shown that the fifth finger is the most common site of phalanx fractures in children [14–16]. Similar results were obtained in the present study. Fifth finger fractures were the most common first-level fracture sites. Chew et al. [14] found that proximal phalanx fractures were more prevalent than distal phalanx fractures. In the school children and adolescents included in this study, the most common second-level fracture site was the proximal phalanx of the fifth finger (65, 22.57% and 45, 29.61%, respectively), which is consistent with the results of Chew et al. [14] However, Rajesh et al. [21] reported that distal phalanx fractures were more common in children aged 0–8 years. In the infants and preschool children included in this study, the most common second-level fracture site was the distal phalanx of the third finger (55, 23.50% and 73, 15.57%, respectively). Therefore, controversy remains regarding whether phalanx fractures in children tend to occur in the proximal or distal phalanx, which may be related to the child’s age. Moreover, because the fifth metacarpophalangeal joint is relatively fragile [22, 23], fractures near the joint are prone to occur after stress, resulting in a high incidence of fifth-line fractures. This phenomenon prompted us to analyze the number of fifth-line fractures (*n* = 326), which included 115 fractures of the proximal phalanx of the fifth finger and 21 fractures of the distal portion of the fifth metacarpal, equaling 136 fractures that accounted for 41.72% of all fractures. This result is similar to that of the study by Worlock and Stower [24], suggesting that children must protect this vulnerable part when engaging in fist-fighting movements. In addition, the first finger aids in fine pinching and grasping, and the first and second fingers coordinate to perform the functions of grasping, indicating, and sensing [25]. Therefore, these fingers are essential for hand function. When these two fingers are fractured, it can greatly affect a child’s hand function. We found that the proportions of first and second finger fractures in infants, preschool children, school children, and adolescents were 29.07, 31.77, 20.51, and 18.10%, respectively. These findings highlight the need for preventive measures to protect the hand function of children.

### Fractures of the foot

Previous studies have shown that most foot fractures in children occur in the metatarsal bones [26], but the most commonly injured metatarsal has not been reported. Among the 418 ft fractures analyzed in this study, 203 (48.56%) occurred in the metatarsal, and most commonly in the second metatarsal (46, 11.00%). Studies have also reported that midfoot and hindfoot fractures occur rarely [27], because greater forces are required; our results are consistent with this finding. Only eight fractures occurred in the tarsus, including five in the midfoot, two in the cuboid bone, three in the lateral cuneiform, three in the hindfoot, and three in the calcaneus. Rammelt et al. [27] believed anatomical reduction is particularly important for fractures of the first and fifth toes; otherwise, it may easily lead to developmental delays or early osteoarthritis.

Denning [10] reported that when the first toe has an intra-articular fracture, the risk of post-traumatic arthritis is higher. When a phalanx fracture is accompanied by severe flexion and extension displacement, malunion is prone to occur. In this study, the number of fractures in the first and fifth toes was relatively high (83, 19.86% and 38, 9.09%, respectively), suggesting that special attention should be paid when treating fractures of these two toes to prevent complications that affect the weight-bearing and balance functions of the toes. In addition, children of different ages have different fracture-prone locations. This may reflect the biomechanical properties of different bones and differences in risk exposure due to age. Further research is needed to explore the biomechanical, growth, and developmental factors behind this difference.

Collectively, these data provide valuable information for the prevention of foot fractures. Targeted protective measures may be required at different stages of a child’s growth. Clinicians should also consider the age distribution of individuals when assessing the fracture site. Future studies should further explore the biomechanical and socio-environmental factors affecting fractures in children of different ages and how to reduce the risk of foot fractures through interventions.

### Etiologies of the injuries

This study revealed the distribution and changing trends in the etiologies of hand and foot fractures in children of different ages. The main etiologies of injuries were daily life injuries; however, the incidence of sports injuries gradually increased with age. These results have important implications in the prevention and management of fractures in children.

Clamping and crushing injuries are usually related to the fingers being caught in doors while playing. The trauma is relatively severe and multiple accompanying injuries may occur simultaneously. In severe cases, dissociative injuries may also occur [19]. Children of different ages may suffer from pinching and crushing injuries that are particularly prominent in infants and preschool children. This study identified 187 cases (75.71%) of pinching and crushing injuries in infants and 334 cases (60.84%) in preschool children. Studies have shown that most finger door entrapment injuries occur at home, particularly in living room and bathroom doors, whereas door entrapment injuries in kitchens are the least common [19, 28]. Therefore, parents should take protective measures, such as installing door stops, which can effectively reduce the incidence of door-clipping injuries [29].

Falls occurred in children of all ages; however, the proportion of falls gradually increased with age. The results for the infants, preschool children, school children and adolescents were 2.83, 12.57, 32.85, and 42.41% respectively. Young children are usually in the early stages of physical development and are more likely to lose balance while walking, running, or playing, resulting in falls. Although older children have a better sense of balance, their activity levels increase accordingly and may involve more outdoor activities [30], which increases the risk of injuries due to falls.

In this study, the proportions of cuts in the above four groups were 12.55, 11.66, 10.95, and 3.16%, respectively. Although relatively uncommon, cuts are an etiology of hand and foot fractures in children. Cuts are often associated with the improper use of knives or other sharp objects while children are playing. To prevent such cuts, parents should ensure that these items are kept out of reach of their children.

Sports injuries are the most common among adolescents [31]. In this study, the number of sports injury cases among adolescents reached 42 (26.58%), much higher than that in the other three groups, which is consistent with the results of previous studies. As children age, they participate in more sports activities, such as basketball, football, skateboarding, and other high-risk activities. Sports involve collisions, falls, and other potentially dangerous situations that increase the risk of hand and foot fractures. This highlights how important it is for children to wear protective gear when participating in high-risk sports and that parents should provide appropriate safety supervision for children.

### Accompanying injuries

The current results showed that hand and foot fractures in children were often accompanied by a variety of injuries, including nail bed, growth plate, distal severed, nerve, vascular, tendon, and joint capsule injuries, as well as compartment syndrome. These complications increase the complexity of the treatment and may lead to long-term functional disability. In this study, nail bed injury was the most common accompanying injury, with 570 cases. Damage to the nail bed affects nail appearance and may lead to permanent growth abnormalities. Restoration of the nail bed requires careful manipulation to restore its original structure and function [32]. Distal severed injury refers to the separation of the distal bone segment from the phalanx. The severed part often exhibits obstruction in the blood supply. When the distal portion is ischemic and necrotic, only a thenar skin flap transfer can be performed, resulting in permanent deformity and loss of finger or toe function. In this study, there were 167 cases of distal severed injuries, 100 of which were caused by clipping injuries. Therefore, to prevent accidental injuries in children, special attention should be paid to avoid clipping injuries.

Nerve and blood vessel injuries can cause sensory abnormalities and circulatory disorders. When the fingers and toes are fractured, the nerves and blood vessels are more likely to be damaged because the soft tissue provides less protection, and the nerves and blood vessels are near the bone. The diagnosis and treatment of such injuries require a comprehensive consideration of the anatomical structure and functional reconstruction. Tendon and joint capsule injuries may result in limited joint motion, decreased strength, or instability [33]. Treatment requires surgical repair or conservative treatment depending on the type and severity of the injury. There are 10 compartments in the hand [34] and nine in the foot [10]. Compartment syndrome is caused by high-energy trauma and crush injuries. Its typical symptoms are pain, pallor, paresthesia, pulselessness, and paralysis. Under anesthesia, when the pressure in any of the foot’s fascial compartments is greater or less than 30 mmHg but lower than the patient’s diastolic blood pressure, fasciotomy and decompression should be performed immediately [35]. This method involves making two longitudinal incisions on the dorsum of the foot and one medial incision along the arch [35]. The hand method involves making two to three longitudinal incisions on the volar or dorsal side [36].

### Growth plate injuries

The epiphyseal plate is a characteristic feature of bones in children. As the epiphyseal plate is mainly composed of cartilage [37], it is easily damaged. Injury to the epiphyseal plate may result in growth arrest or deformity [38]. In this study, there were 296 cases of epiphyseal plate injuries, 246 of which occurred in the hands and 50 in the feet, accounting for a considerable proportion of the 1561 fractures. The results indicated that the fifth finger was the most common site of epiphyseal fractures, and most occurred in the proximal phalanx of the fifth finger, making the fifth metacarpophalangeal joint highly susceptible to epiphyseal fractures. Therefore, children should avoid high-intensity hitting movements to reduce the risk of epiphyseal fractures of the fifth metacarpophalangeal joint. Among the growth plate injuries of the foot, the first toe was the most common site, being fractured more often than the other four toes combined. In addition, growth plate injuries of the proximal phalanx were the most common, whereas those of the middle phalanx were the least common. Therefore, these results suggest that clinicians should focus on examining the predilection sites when evaluating epiphyseal plate injuries.

The aforementioned accompanying injuries require individualized treatment on a case-by-case basis. Timely and accurate diagnosis, appropriate treatment, and subsequent rehabilitation management are crucial for restoring hand and foot functions and preventing disability. The prevention and treatment of accompanying injuries is a multidisciplinary and multifaceted challenge that requires collaboration among orthopedics, rehabilitation, neurosurgery, and other disciplines. We recommend comprehensive treatment under the guidance of a professional orthopedic surgeon to achieve the best treatment results.

This study had some limitations. First, this was a single-centre study and lacked multicenter data to support the generalizability of the findings. Second, data on concomitant injuries were extracted from medical records and their definitions lacked uniform standards.

## Conclusion

Overall, this study provided detailed data on hand and foot fractures in children, including information on sex, fracture site, etiology, and accompanying injuries, and analyzed age trends in the fracture sites and etiologies of injuries in children. These data may aid medical personnel in better managing hand and foot fractures in children, improving prevention, treatment, and rehabilitation methods and providing valuable information for developing effective management strategies.

## Data Availability

All data generated or analyzed during this study are included in this published article and its supplementary information files.
